# Evaluating the subject-performed task effect in healthy older adults: relationship with neuropsychological tests

**DOI:** 10.3402/snp.v5.24068

**Published:** 2015-04-10

**Authors:** Ana Rita Silva, Maria Salomé Pinho, Céline Souchay, Christopher J. A. Moulin

**Affiliations:** 1 Faculty of Psychology and Sciences of Education, University of Coimbra, Coimbra, Portugal; 2 Laboratoire d'Etude de l'Apprentissage et du Développement, LEAD-CNRS (UMR 5022), University of Bourgogne,, Dijon, France

**Keywords:** aging, subject performed task, memory for actions, episodic memory

## Abstract

**Background:**

An enhancement in recall of simple instructions is found when actions are performed in comparison to when they are verbally presented – the subject-performed task (SPT) effect. This enhancement has also been found with older adults. However, the reason why older adults, known to present a deficit in episodic memory, have a better performance for this type of information remains unclear. In this article, we explored this effect by comparing the performance on the SPT task with the performance on other tasks, in order to understand the underlying mechanisms that may explain this effect.

**Objective:**

We hypothesized that both young and older adult groups should show higher recall in SPT compared with the verbal learning condition, and that the differences between age groups should be lower in the SPT condition. We aimed to explore the correlations between these tasks and known neuropsychological tests, and we also measured source memory for the encoding condition.

**Design:**

A mixed design was used with 30 healthy older adults, comparing their performance with 30 healthy younger adults. Each participant was asked to perform 16 simple instructions (SPT condition) and to only read the other 16 instructions (Verbal condition – VT). The test phase included a free recall task. Participants were also tested with a set of neuropsychological measures (speed of processing, working memory and verbal episodic memory).

**Results:**

The SPT effect was found for both age groups; but even for SPT materials, group differences in recall persisted. Source memory was found to be preserved for the two groups. Simple correlations suggested differences in correlates of SPT performance between the two groups. However, when controlling for age, the SPT and VT tasks correlate with each other, and a measure of episodic memory correlated moderately with both SPT and VT performance.

**Conclusions:**

A strong effect of SPT was observed for all but one, which still displayed the expected aging deficit. The correlations and source memory data suggest that the SPT and VT are possibly related in respect to their underlying processes, and SPT, instead of being an isolated process, is in connection with both the episodic memory and executive function processes. Under these circumstances, the SPT seems to contribute to an enhancement of the episodic memory trace, presumably from the multimodality it provides, without involving a separated set of cognitive mechanisms. Future research using more pure measures of other cognitive processes that could be related to SPT is necessary.

In the early 1980s, Engelkamp and Krumnacker (1980) and Cohen (1981) introduced a new paradigm in the study of memory, the SPT paradigm (subject-performed task). From this paradigm, they proposed fundamental differences between episodic memory for verbal information and episodic memory for the prior execution of actions. They proposed that the differences in people's performance when confronted with these tasks resulted from the mechanisms used in this kind of information processing (Zimmer & Cohen, 2001). Subsequently, many studies have replicated the basic effect that memory is enhanced when the subject enacts to-be-remembered materials. Various proposals have been made as to why there is a difference between verbal information and the execution of actions. Some researchers stress the role of the motor and multimodal information that strengthens the memory trace (Engelkamp & Krumnaker, 1980; Engelkamp & Zimmer, 1983, 1985), whereas other researchers highlight the processing of actions as being a more automatic process (non-intentional), which is thus easier to retain and to recall than verbal information (Cohen, 1983).

A critical issue in the SPT effect is automaticity. Cohen (1983) suggested that the processing involved in the SPT is automatic, therefore, not requiring effort or strategy. They observed other concomitant peculiarities of the SPT effect, such as the absence of a primacy effect in the serial position curve [a point also noted by Helstrup (1986)], the lack of an effect of elaboration of the items (e.g. no differences in the recall of sentences with more than one object, or with more than one action included) and the presence of a similar effect regardless of the list size [data confirmed later by Nilsson and Cohen (1988)]. These phenomena are clearly different from those observed in tasks of verbal learning and led to the conclusion that processing in tasks of memory for actions is governed by memory processes different from those in verbal and visual episodic memory. Cohen developed this idea to suggest that this non-strategic process underlying the SPT effect would diminish the executive load related to the task, thus being easier for people with some executive impairment, such as older adults. However, Engelkamp and Cohen (1991) pointed out that this nonstrategic theory is an argument that has already been questioned by other researchers. For example, Kausler (1989) showed that the more cognitively demanding the task was, the more age differences were found in the SPT tasks, which goes against the theory of automaticity.

Defending another perspective, Engelkamp and Krumnaker (1980), Engelkamp and Zimmer (1983), and Engelkamp and Zimmer (1985) focused on the role of the motor component in performance, indicating that the motor execution of the verbal instructions is responsible for the SPT effect. In support of this theory, Engelkamp and Zimmer (1989) observed the performance of participants in the SPT condition, an imagination condition and also an EPT condition (the participant observed the experimenter performing the action). The result was found that both the imagination and the EPT conditions exhibited significantly lower scores than the SPT condition, which led them to conclude that something more than the mental image or the observation of the actions is involved in the effect (Engelkamp, 1995). These researchers propose that the motor activation code (produced by enactment) optimizes the item specific information integrating the object with a specific action (Engelkamp & Zimmer, 1989). Further, Bäckman and Nilsson (1984, 1985) proposed that enactment during encoding produced a richer multimodal storage, which joined the characteristics of objects or events (color, texture, shape, size, etc.). Thus the semantic, perceptual and motor information which is not encoded in the verbal (control) condition, but which is integrated into the memory trace in the SPT condition explains the SPT effect (Steffens, 2007, Zimmer, Helstrup, and Nilsson, 2007). However, this multimodality theory is not without criticism. Nyberg, Nilsson & Bäckman (1992) found that the objects and their sensory richness were not critical for the SPT effect: the enactment of the action alone is sufficient to provide item-specific information with or without the presence of the object (see also Engelkamp & Cohen, 1991). An approach that helps us to understand the effect of enactment in memory is the research conducted into gestures (for a review, see Madan & Singhal, 2012a), where it was found extensively that mere gesturing enhanced learning and memory, again suggesting that the presence of the object is not necessary to produce the SPT effect. Madan & Singhal (2012b) also showed that even the motoric properties of the to-be-remembered, such as their movement-related properties, affects memory recall, such that the more manipulable an object or the more movement imagery produced by the words to be recalled, the easier it is to recall that information. So the ‘movement component’ of the information is apparently critical in explaining the enhancement of memory, in agreement with what Engelkamp and colleagues first hypothesized.

The focus of this article is healthy aging, where there is a well-documented deficit in episodic memory, such that older adults show reduced recall and particular difficulty in tasks that require intentional effort (Earles & Kersten, 2002; Naveh-Benjamin, 2000). Perhaps the clearest manifestation of this deficit is the ‘associative-deficit hypothesis’ of cognitive aging. This paradigm suggests that older adults have difficulty in integrating the multiple, related and unrelated features of an episode (Naveh-Benjamin, 2000; Naveh-Benjamin, Hussain, Guez, & Bar-On, 2003). Many researchers, employing a variety of different stimulus–stimulus pairings, have shown that older adults demonstrate relatively intact recognition for individual items while presenting impaired recognition of the association between them (Castel & Craik, 2003; Naveh-Benjamin, 2000; Naveh-Benjamin, Kilb, & Reedy, 2004; Naveh-Benjamin et al., 2003).

Bäckman and Nilsson (1984) were the first to use the SPT paradigm with older adults. Eight lists of 12 simple instructions each were presented to a sample of 36 young adults and 36 older adults, with half of the lists presented in the SPT condition (the participants performed the simple instructions) and the remaining lists presented in the control condition in which participants only had to read the instructions. For both age groups, superior free recall was found in the SPT condition. Furthermore, it was found that the difference between groups, although not eliminated, decreased significantly in the SPT condition. These results led to the idea that older adults benefit particularly from this type of task (Rönnlund, Nyberg, Bäckman, & Nilsson, 2003). Rönnlund et al. proposed that the presence of multimodal cues eliminates older adults’ difficulties in auto-initiated processes of remembering (see also Steffens, 2007).

Brooks and Gardiner (1994) repeated Bäckman and Nilsson's (1984) study but included two lists of 16 sentences each, and in each list, half of the sentences concerned familiar activities and the other half included less common actions. The results confirmed the findings of Bäckman and Nilsson (1984), in that there was a significant decrease in the difference between young and old adults (almost to the residual level) in the SPT condition, regardless of the familiarity of the actions (Nilsson, 2005). Nyberg, Nilsson, and Bäckman (1992) attempted to examine to what extent this effect of superior performance under the SPT condition is increased in older adults, if, in the recall phase, the participants were prompted to perform, again, the actions they could remember. However, they found that this method did not significantly improve the performance of both groups in SPT condition.

Norris and West (1993) analyzed the SPT effect in a sample of 80 young adults and 80 older adults, where two lists were presented, each with 16 statements. In one of the lists, items could be organized into semantic categories, while in the other there was no semantic grouping of items. It was found that differences between groups were maintained for verbal and SPT conditions, despite there being superior performance in the SPT condition for both groups. Additionally, it was observed that the group of older adults benefited more from the organized list than the young group, even in the SPT condition. This finding led to the reflection that the characteristics of the list as well as its organization may influence the differences between groups, consequently meaning that there is no pure SPT processing, e.g. there is no independent cognitive processes involved in coding actions (Norris & West, 1993). Earles, Kersten, Mas, and Miccio (2004) also concluded that when there is a time limit to perform the SPT task, older adults’ performance is diminished suggesting that in these cases the presence of the object and consequently the overload of the visual system affects their performance.

Earles (1996) studied the SPT effect on a sample of 101 young adults and 101 older adults, analyzing memory for brief sentences enacted (four lists of 12 SPTs) and for non-enacted items (four lists with 12 specific names). In this within-subjects design, the SPT effect was observed for both groups in memory of enacted sentences, although the recall for the group of young adults was significantly higher than for the group of older adults. Also McDonald-Miszczak, Hubley, & Hultsch (1996) concluded that, although the enactment of information improves recall, regardless of age, this benefit is not enough to reduce the differences between the groups. The theoretical issue of whether the age difference is reduced or removed in older adults pertains to the basis of the effect. If older adults show a deficit on one task, but not on the other, the neuropsychological rationale might be that the two processes are independent.

Rönnlund and collaborators (2003) analyzed the memory performance in an action memory task, using a sample of 1,000 subjects aged between 35 and 80 years of age, divided in 10-year age groups. The participants had to study two lists of 16 simple instructions. The results of free recall showed a main effect of age and condition. Additionally, there was a decrease in differences between the age groups in the SPT condition, although a more thorough analysis, suggested that there was a possible floor effect in verbal learning for older age groups, which may explain this pattern of results.

Mangels and Heinberg (2006) studied the SPT in older adults as a potential promoter of a broader episodic integration. For this purpose, they observed the performance of 30 young adults and 30 older adults in a memory for actions task which included four lists with 20 instructions each. Two types of lists were created, lists with action – object pairs semantically related (e.g. *put the phone to the ear*) and lists with unrelated object – action pairs (e.g. *shake the phone*). The aim of manipulating the semantic association was to examine whether the associative potential underlying the SPT effect would be independent of the semantic information of items. Their results showed a superiority for SPT encoding compared to verbal encoding, for both groups, with, however, a superior performance for the group of young adults in comparison with the older adults. In the SPT task there were no differences in performance for the lists in any of the groups, which indicates that this type of effect overcomes any semantic contribution. Taken together, these results led the researchers to conclude that the execution of sentences provides a benefit to forming associations, even for a group (the older adults) that usually have difficulties in associating information, even if this effect cannot completely eliminate the performance differences between this group and young adults (Mangels & Heinberg, 2006).

In summary, there is overwhelming evidence that older adults show, at the least, a SPT effect which is parallel to that seen in younger adults (for a review see Feyereisen, 2009). Furthermore, studies with clinical populations have been conducted, showing that this benefit even extends to people with degenerative diseases such as Mild Cognitive Impairment (Karantzoulis, Mangels, & Rich, 2006) or Alzheimer's disease (Dick, Kean, & Sands, 1989; Fairfield & Mammarella, 2009; Lekeu, Van der Linden, Moonen, & Salmon, 2002; Mack, Eberle, Frolich, & Knopf, 2005), transient global amnesia (Hainselin et al., 2014) and other neurological conditions (Knopf, Mack, Lenel, & Ferrante, 2005) (for a review see Hainselin, Quinette, & Eustache, 2013). However, there is fewer consensuses regarding the locus of this effect and the results of studies into older adults are marked by strong differences in the magnitude of the SPT effect. Almost all of the studies presented with non-convergent results about the nature of the SPT effect in aging follow different methodologies, both with regard to the list characteristics and the experimental design used (Lekeu et al., 2002). The issue of whether the effect reduces or removes the episodic deficit (and therefore whether it might be linked to the same system or derive from a different process in episodic memory) is in question. Thus far, there has been less emphasis on whether older adults know the basis of their improved memory (can they accurately report the source of the to-be-studied as enacted or not) and whether SPT and other forms of episodic memory are related.

The present study had the aim of looking at performance on other memory and cognition tasks to examine correlates of the effect, with a view to making a small but specific contribution to the aging literature. We can possibly begin to disentangle explanations about the underlying cognitive processes involved in memory for actions by seeing if related tasks converge on the same processes. For instance, is intact executive function required to show the SPT effect? Is it those people with better episodic or working memory measures who show more of a benefit in SPT processing? Previous studies have focused on the SPT task compared to a Verbal task, but no information is described about the performance of the participants in other cognitive domains that could allow us to make some associations between the SPT processing and other cognitive processes. Let us imagine that there is a correlation between SPT performance and a different, standardized episodic memory test, for instance. This would suggest that the effect was in some way driven by, or at least reliant upon a level of episodic memory function. If the effect is automatic, on the other hand, or perhaps driven by motoric processes not captured in measures which classically correlate with episodic memory in aging, it might suggest that the SPT effect bypasses those processes implicated in the episodic memory deficit in older adults: executive function, speed of processing, working memory (for review see Balota, Dolan, & Duchek, 2000). Moreover, measurement of sub components of memory, such as source memory, also may help us elucidate the basis of the SPT effect. Source memory is typically impaired in older adults (Glisky, Rubin, & Davidson, 2001), which is typically seen as an extension of the failure to make associations at encoding which are later useful in retrieval, since the older adult just remembers that he has seen the item and not in what context. If SPT instructions enhance the retrieval of source, and indeed if older adults can reliably report how they encountered materials in SPT and verbal conditions, it will point to the fact that the SPT instructions enhance and compliment this facet of episodic memory (memory for source) rather than by-pass it.

Thus, the present study used the SPT paradigm with older adults, and a number of other measures in an exploratory fashion. If the explanations stressed by Cohen and Engelkamp about the possible underlying mechanism of processing actions are true, older adults should show a strong SPT effect, because the processing is effortless (Cohen, 1983) and because the multimodal information provided with enactment (Bäckman & Nilsson, 1985; Engelkamp & Zimmer, 1989) strengthens the memory trace. We will compare older adults’ performance in SPT with their performance in tasks where they normally show some impairment: working memory, speed processing, source memory, and verbal episodic memory. This will help us to understand if the SPT effect is independent of cognitive domains that are more exposed to disruption through life span, thus being a useful paradigm to introduce in cognitive stimulation of this group.

## Method

### Subjects

Participants were recruited in the community, home care centers and senior universities. A total of 60 people participated: 30 young adults (age range: 35–45 years; *M*=41.43, SD=2.73) and 30 older adults (age range: 70–75 years; *M*=72.30, SD=1.62), all of whom had normal or corrected vision and hearing, and were not exhibiting significant depressive symptoms. The older participants also did not show cognitive decline, according to the Addenbrooke's Cognitive Examination-Revised instrument, a 15-min screening test with a range of tasks to detect cognitive deficits (this includes tests of Orientation and Attention, Memory, Verbal Fluency, Language and Visuo-Constructive Capacity; and that incorporates the known screening test Mini-Mental State Examination; MMSE: *M=*28.53, SD=.97; ACE-R: *M*=84.87, SD=5.67). The groups did not differ in terms of gender, education, premorbid IQ and dwelling area (Table 1). All participants provided informed consent, and all study procedures were approved by the research ethics board of the faculty.

**Table 1:** Demographic and general cognitive characteristics of participants

*N=*60	Younger adults *N=*30	Older adults *N=*30
Age	*M=*41.43 (*DP*=2.72; *A*=35–45)	*M*=72.30 (*DP=*1.62; *A*=70–75)
Gender	Female=23 (76.7%) Male=7 (23.3%)	Female=24 (80%) Male=6 (20%)
Education	4 years=20 (66.7%) 6 years=10 (33.3%)	4 years=23 (76.6%) 6 years=7 (23.3%)
Dwelling area	Urban=2 (6.7%) Rural=28 (93.3%)	Urban=5 (16.7%) Rural=25 (83.3%)
Vocabulary (estimated IQ measure)	*M*=31.00 (*DP*=5.68)	*M*=33.13 (*DP*=7.39)
MMSE (0–30)		*M*=28.53 (*DP*=.97)
ACE-R (0–100)		*M*=84.87 (*DP*=5.67)
GDS (0–30)		*M*=6.40 (*DP*=2.896)
BDI (0–63)	*M*= 5.33 (*DP*=2.26)	

### Materials

#### Memory for actions test

A Memory for Actions Task was created for this study based on the SPT paradigm (Cohen, 1981, 1983; Engelkamp & Krumnaker, 1980). This task comprised 34 sentences (32 plus 2 for training trials) related to simple and familiar actions (e.g. ‘throw the dice’), with each sentence written in black on a white card; and 34 familiar objects (32 plus 2 for training trials). Two lists with 16 sentences each (plus 1 for training) were created. While showing each pair (sentence and object) to the participants, the experimenter read the sentence out loud. For one of the lists the participants only read the sentences (VT–verbal condition) after hearing the experimenter, and for the other list the participants were asked to perform the action in the presented sentence (SPT condition). Each pair was shown for 12 seconds after which the object and sentence were hidden from the participant's view. Participants were told that later in the session they would be asked to recall the sentences. After a brief distractor task (counting backwards for 40 seconds), the participants recalled the sentences presented under both encoding conditions, for 5 min.

To assess source memory, a task usually described to show a poor performance by older adults (Glisky et al., 2001), all sentences were randomized and read by the experimenter and the participants had to answer verbally if each sentence had been enacted or not. Proficient source memory is shown by being able to correctly report which condition the sentence had been encountered in.

This experiment followed a within-participants design. The sentence coding was in two blocks of 16 sentences each, according to the sequence VT–SPT or SPT–VT. The order of each sequence was counterbalanced (half participants did the VT–SPT sequence and the other half did the SPT–VT sequence) and each sentence was coded the same number of times in both conditions.

#### Complementary neuropsychological measures

We aimed to contribute to the debate about the underlying cognitive processes involved in the SPT effect. For this reason we constructed a set of neuropsychological tests, which was administered to all participants, including measures of speed of processing, attention, working memory and verbal episodic memory. The assessment included measures that are known to show a relative decline in cognitive aging as we intended to understand the singularity of the SPT effect in aging compared to these cognitive domains. A short description of the tests and the cognitive domains assessed by each test is described below:

1. *Symbol Search* (Wechsler Adult Intelligence Scale-III; WAIS-III; Wechsler, 1997, 2008) is a task that analyses the speed of processing and attention (both sustained and selective).
2. *Digit Symbol-Coding* (WAIS-III; Wechsler, 1997, 2008) is a task that tests speed of processing.
3. *Portuguese version of WMS Verbal Paired Associates subtest* (Wechsler Memory Scale-III, 2008), this tests episodic memory of five semantically related pairs – like ‘Boat-River’ – and five non-related pairs – like ‘Offer-Speed’. Data from immediate recall and delayed recall can be obtained from this test.
4. *Visual Patterns Test* (Della Sala, Gray, Baddeley, & Wilson, 1997). This is a task that examines visual working memory performance and visual short-term memory. As an extended literature suggests this task is predicted by visuo-spatial executive function capacity (Barrouillet & Camos, 2010; Brown, Brockmole, Gow, & Deary, 2012; Fournier-Vicente, Larigauderie, & Gaonac'h, 2008; Miyake, Friedman, Rettinger, Shah, & Hegarty, 2001), for the purpose of interpreting the results we considered this task a measure of executive functioning.
5. *Letter-Number Sequencing* (WMS-III; Wechsler, 1997, 2008) is a test used to assess verbal working memory performance, sequential processing and memory span. Because this task involves complex item manipulation, it is also interpreted in this study, in agreement with literature, that this is as a measure of executive function (Emery, Myerson, & Hale, 2007; Salthouse, 2005; Myerson, Hale, Rhee, & Jenkins, 1999). Based on the multi-components model of Baddeley & Hitch (1974) and Baddeley (2000), working memory includes a central component described as the ‘central executive’.

## Results

The results section is organized as follows: firstly, we will present data from the SPT Task, both the total recall and the source memory measures; secondly, we will present data from the complementary neuropsychological measures for both groups; and finally, we will present the correlations for the two age groups between the Memory for Actions task conditions and the neuropsychological measures.

### Memory for actions task

#### Total recall

The correct recall of the complete sentences (correct action–object pair) was analyzed in both age groups, in the VT and the SPT conditions. A mixed ANOVA with repeated measures for condition (VT versus SPT) was performed to analyze the differences between the two age groups. From this analysis, the expected main effects of group [*F*(1, 58)=41.24, *p*<.001, η^2^_*p*_=.416] and condition were found [*F*(1, 58)=470.39, *p*<.001, η^2^_*p*_=.890]. Moreover, with these participants and with this design, an interaction between group and condition was found [*F*(1, 58)=9.61, *p=*.003, η^2^_*p*_=.142], where the differences in total recall between groups diminished in the SPT condition (in this condition the performance of both groups was higher (young adults *M*=.57, SD=.13; older adults *M*=.47, SD=.08) than in the verbal condition (young adults *M*=.35, SD=.09; older adults *M*=.17, SD=.08). However, when going further into this analysis, a paired samples *t*-test for each group between the SPT and VT found significant differences [Younger Adults: *t*(1, 29)=−11.80, *p*<.001; *d=*2.17; Older Adults: *t*(1, 29)=−20.11, *p*<.001; *d=*3.67]. Likewise, we found significant differences when we analyzed the group differences in SPT and VT separately, in an independent sample *t*-test [SPT: *t*(1, 58)=3.81, *p*<.001; *d*=3.79; VT: *t*(1, 58)=8.02, *p*<.001; *d*=8.08], thus suggesting that although the SPT increased memory performance in the older adults, significant differences remained between the young and old, which is clear in the large effect sizes of these analyses.

#### Source memory test

All 32 sentences were re-presented to the participants, and they reported in which condition they had encoded the materials. A one-way ANOVA showed no differences between groups in source recognition: *F*(1, 58)=1.98, *p=*.164. Interestingly, both the younger adults group (*M*=30.90; SD=.92) and the older adults group (*M*=30.50; SD=1.25) correctly recognized the source for almost all the sentences (Max=32). However, because of the evident ceiling effect with this task, we must be cautious regarding the apparent lack of differences between groups in source memory for the Memory for Actions Test.

When looking at the errors (very few as we can see by the recognition scores) committed by participants, we separated the errors committed for sentences coded in the SPT condition and in the VT condition, and we performed a repeated measures ANOVA for these errors within conditions and between age groups. An interaction effect was found between group and the type of error [*F*(1, 58)=9.079, *p*=.004], suggesting that the groups differ less in errors for sentences coded under enactment (Younger Adults *M*=.33, SD=.547; Older Adults *M*=.17; SD=.461) than for sentences coded under the verbal condition (Younger Adults *M*=.77, SD=.728; Older Adults *M*=1.33; SD=.17), although the effect size for this interaction can be considered weak (η^2^=.135).

#### Differences in cognitive performance between young and older adults

Table 3 shows the expected pattern of deficits in older adults on our cognitive task measures, with only the difference in digit symbol coding (incidental learning) showing a non-significant difference between groups. Thus far, we can therefore characterize our older adult group as a group who show declined performance in long term memory and executive function, and who equally have a decline in the verbal and SPT components of our experimental measures. Nonetheless our older adult group has shown a strong SPT effect, and therefore we have replicated the known patterns of data in this domain. Of interest, when asking older adults about their memory function, they show a near-perfect source memory for their actions. In this dichotomized decision, older adults, as their young counterparts, can reliably report how they encountered materials in a later recognition task, even if they have not been able to recall previously the sentence including the object and action. The final section of this methods section considers a set of individual differences analyses to explore correlates of SPT performance (Table 2).

**Table 2:** Means and statistical tests for young and older adult groups, neuropsychological measures

	Younger adults		Older adults				
				
	Mean	SD	Mean	SD	*t*	Sig.	Cohen's *D*
Symbol Search (WAIS-III)	17.37	3.94	11.40	3.49	6.206	.000*	1.60
Digit Symbol Coding – codification	48.77	8.42	25.13	4.73	13.398	.000*	3.46
Digit Symbol Coding – incidental learning	11.03	3.62	10.20	4.30	.812	.420	.20
Coding – free memory task (WAIS-III)	7.93	.98	7.37	.85	2.392	.020	.61
Coding – copy (WAIS-III)	78.53	20.79	52.87	13.64	5.653	.000*	1.46
Letter–Number Sequencing (WMS-III)	10.60	1.87	7.03	1.33	8.528	.000*	2.20
Visual Patterns Test	5.04	.69	3.74	.41	8.918	.000*	2.29
WMS Paired Associates Test – immediate	13.50	4.62	10.00	3.52	3.299	.002*	.85
WMS Paired Associates Test – delayed	6.10	1.49	4.17	1.21	5.516	.000*	1.42

* sig<.001.

### Relation of the performance in the memory for actions task and the neuropsychological measures

First we present the simple correlations between the two conditions of the Memory for Action task (VT and SPT conditions) and the neurocognitive measures for each group separately (see Table 3). To control for the number of correlations conducted we performed the Benjamin and Hochberg False Discovery Rate correction (1995) as a multiple comparison procedure to correct the significance scores. In the young group, for the VT condition, we find positive and strong associations with the immediate task of the WMS Paired Associates test [*r*_*s*_(30)=.735, *p*=.01], and also with the Letter–Number Sequencing [*r*_*s*_(30)=.780, *p*=.01]. Additionally, we found a positive, moderate association between the VT condition and the Symbol Search test [*r*(28)=.469, *p=*.04]. For the SPT condition in the young group, a different pattern of associations was observed; positive, but weak to moderate associations with only two tests of executive function and attention: Visual Patterns Test [*r*(28)=.473, *p=*.04]; Symbol Search [*r*(28)=.571, *p*=.02]. Analyzing the correlation matrix for the older adults group, we firstly see fewer significant correlations. In the VT condition, we identified positive and weak to moderate associations with the WMS Paired Associates test immediate [*r*_*s*_(30)=.446, *p*=.05], and with the Digit Symbol Coding [*r*_*s*_(30)=.486, *p=*.04]. In the SPT condition, we found no statistically significant associations with any of the neurocognitive tests used.

**Table 3:** Correlation matrix between VT and SPT Conditions and the Neuropsychological tests, for the young and older adult groups

		WMS Paired Associates Test – immediate	WMS Paired Associates Test – delayed	Letter–Number Sequencing	Visual Patterns Test	Digit Symbol Coding – codification	Symbol Search
Younger adults	VT Condition – Memory for Actions	.735	.304	.780	.311	.292	.469
		*p*=.01	*p*=.17	*p*=.02	*p*=.17	*p*=.18	*p*=.04
	SPT Condition – Memory for Actions	.335	−.066	.416	.473	.376	.571
		*p*=.14	*p*=.86	*p*=.08	*p*=.04	*p*=.11	*p*=.02
Older adults	VT Condition – Memory for Actions	.446	.407	.410	−.047	.486	.028
		*p=*.05	*p=*.13	*p=*.13	*p=*.86	*p=*.04	*p=*.89
	SPT condition – Memory for Actions	.236	.135	.041	.045	.212	−.333
		*p=*.25	*p=*.61	*p=*.86	*p=*.86	*p=*.35	*p=*.14

If there is some pattern in these correlations, it is that the verbal condition of our experimental task seems to show a moderate to strong association with our test of episodic memory, the Paired Associates Test. This pattern is clearer in the immediate test in the younger and older adults. On the other hand, the paired associates’ measure does not correlate significantly with the SPT task in either group.

To elucidate further the role of our other cognitive measures on the SPT, we considered an analysis combining the groups but controlling for age with partial correlations. Like this, we aimed to examine if there were any common factors which influenced the SPT. That is, we had group differences in SPT and most of our neuropsychological measures, so it is difficult to know what neuropsychological measures influence directly the SPT and which may just be part of a common aging deficit. In particular we were interested in further examining the above trend that whereas tests of episodic memory (and a few others beside) are associated performance in the verbal condition, there is a different set of correlations for the SPT task – in particular a lack of the relationship with the paired associates task.

In these partial correlations controlling for age (see Table 4), we observed moderate relations between our neuropsychological measures and experimental tasks, with all but one of our measures (visual pattern test) correlating with both SPT and VT measures. Thus, once having controlled for age, there appears to be no difference in neuropsychological correlates (at least the broad spread that we have chosen) for SPT and VT. Perhaps this pattern is best summarized with the fact that SPT and VT tasks themselves correlate very strongly when controlling for age: those people who do well on one test, do well on the other (*r*=.57, *p*=.01). Finally, as this pattern in partial correlations was not clear enough to understand the relationship between SPT and the other measures, we decided to analyze the groups as a whole, not controlling for age, and our results were that all complementary neuropsychological measures correlated with both the VT and the SPT, and a stronger correlation between the VT and the SPT (*r*=.67, *p*=.01). The episodic memory test (WMS Verbal Paired Associates) correlated more strongly with the VT (Immediate *r*=.70, *p*=.01; Delayed *r*=.75, *p*=.01) than with the SPT (Immediate *r*=.51, *p=*.01; Delayed *r*=.45, *p=*.01) but all correlations were significant. Thus, we might therefore conclude that the SPT task has a relationship with the general episodic memory system, despite it not being entirely driven by it. The two tasks used to measure executive function also correlated positively and strongly with both the VT (Visual Patterns Test *r*=.62, *p=*.01; Letters–Numbers Sequencing *r*=.81, *p=*.01) and the SPT (Visual Patterns Test *r*=.56, *p=*.01; Letters–Numbers Sequencing *r*=.55, *p=*.01), indicating again the role of executive functioning in the SPT. Finally, for the attention and speed processing measures, we find the same results, with similar correlations for both VT and SPT with these measures, but these are stronger for the VT task (Digit–Symbol Coding *r*=.74, *p*=.01; Symbol Search *r*=.61; *p*=.01) than for the SPT (Digit–Symbol Coding *r*=.56, *p*=.01; Symbol Search *r*=.46; *p*=.01).

**Table 4:** Partial correlations between VT and SPT Conditions and the Neuropsychological tests, controlled for age groups

	WMS Paired Associates Test – immediate	WMS Paired Associates Test – delayed	Letter–Number Sequencing	Visual Patterns Test	Digit Symbol Coding - codification	Symbol Search
VT Condition – Memory for Actions	.649	.581	.588	.151	.325	.280
	*p=*.01	*p*=.01	*p*=.01	*p*=.26	*p*=.02	*p*=.05
SPT Condition – Memory for Actions	.404	.267	.355	.371	.385	.260
	*p*=.01	*p*=.05	*p*=.02	*p*=.01	*p*=.01	*p*=.06

Our final analysis considered using a regression to predict the SPT and VT scores. When carrying out the regressions without splitting the age groups, both VT recall and SPT recall are significantly predicted by the combined model of all the other tests together [VT, *R* ^2^=.76, adjusted *R* ^2^=.73, *F*(6, 53)=27.99, *p*<.001; SPT,=.46, adjusted *R* ^2^=.40, *F*(6, 53)=7.61, *p*<.001] (but alone, no isolated test explained a significant proportion of the variance of the results in both conditions). However, when separating the analysis by age groups, one interesting finding was produced, whereby VT recall and SPT recall are predicted by the combined model of complementary neuropsychological measures only for the younger adults [VT, *R* ^2^=.73, adjusted *R* ^2^=.67, *F*(6, 23)=10.70, *p*<.001; SPT, *R* ^2^=.65, adjusted *R* ^2^=.55, *F*(6, 23)=6.96, *p*<.001], whereas for the older adults, both VT results and SPT results are not predicted by the model of complementary tests, neither by any of these tests alone [VT, *R* ^2^=.28, adjusted *R* ^2^=.09, *F*(6, 23)=1.50, *p*=.223; SPT, *R* ^2^=.30, adjusted *R* ^2^=.12, *F*(6, 53)=1.68, *p*=.172]. Perhaps the significant differences in most cognitive measures used in this study between older and younger adults justify this pattern, which we will consider in more detail later in the discussion section.

## Discussion

This study builds on a succession of studies examining the SPT in older adults. Like those before us, we found two clear effects: a deficit in memory in older adults, but a significant SPT effect. If anything, our older adults showed more of an SPT effect than the younger adults, as shown by a significant interaction, but nonetheless, follow-up *t*-tests showed that significant age differences remained in SPT performance. A similar pattern was obtained by Bäckman & Nilsson (1984), Brooks & Gardiner (1994), and Norris & West (1993), Earles (1996) and Mangels & Heinberg (2006), who used a sample and methods with similar characteristics to the present study.

Based on the original studies of the SPT paradigm with older people, we were interested in the possibility that differences between the two age groups were not eliminated in the SPT condition. Additionally, the fact that the VT and the SPT condition correlated with each other in the correlation matrix suggests that performance in these two tasks is related, so probably activating the same substrate of episodic memory, at least partially (since the correlation is only moderate – *r*=.67). This is also supported by the correlations performed not controlling for age, where the SPT task correlated moderately with the episodic memory measures, thus meaning that there is an involvement of episodic memory in the performance of SPT tasks. Thus we have found that a powerful SPT effect exists in older adults, but not one which reduces age differences to non-significant levels.

The novelty of the experiment was also to consider the SPT task alongside a report of source: are people aware of how they studied the material, given that they benefit from performing the action, or is it more implicit than that? Considering source memory, previous studies have suggested a dissociation between item memory and source memory, verifying the benefit of the SPT condition only for memory for what was studied and not in which condition, but this study included no older adults, only young adults (Hornstein & Mulligan, 2004). In our study, we analyzed the source memory in older adults as this age group usually shows deficits in this type of task (McDaniel Einstein & Jacoby, 2008). Here the results obtained by the two age groups for the source recognition showed no differences, and almost all the items learned in both conditions were recognized. As we can observe a ceiling effect for this task, we remain cautious regarding the interpretation of this task. However, on the whole, the results point to older adults being able to report the source of the items studied. When looking at the errors committed in source recognition, both the young and the older adults showed more errors for sentences encoded in the verbal condition (VT) than in the SPT condition. Despite the effect size of this difference being relatively weak, we can suggest that older adults, who present more difficulty in this task normally, are more able to report the source of items when they were encoded by enactment, and this effect apparently helps them to be more aware of source.

We also considered whether a number of standard neuropsychological measures would point to the basis of the SPT effect. We were most interested in whether SPT and VT conditions had different correlates, and in whether episodic memory performance *per se*, measured on another task, correlated with either VT or SPT or both. For the most part, we found strongest correlations in the young group, and for the VT task. However, when considering the group as a whole, it is clear that VT and SPT tasks correlate with each other moderately, and that they also correlate with our other neuropsychological tests. A number of more fine-grained analyses of the simple correlations split by group do point to some interesting patterns which we suggest are interpreted with caution, but which may well be interesting to take forward in future studies. In the first matrix, for instance, we found that, while the older adults’ SPT performance showed no significant correlation with the neurocognitive measures (with moderate correlations with episodic memory and speed processing measures for the VT condition), for the group of younger adults, the neuropsychological tests have positive and moderate relationships with the VT and SPT tasks (despite in the SPT task the correlations are only significant for executive function and attention measures). The crucial test was, moreover, the correlations without controlling by age groups, where the SPT showed positive, moderate and significant correlations with all the neuropsychological measures used in this study (episodic memory, executive function and attention/speed processing), which, despite suggesting lower correlation strength compared to the VT task (that showed strong and positive correlations with all the cognitive measures), indicates that the SPT effect is not a separate type of memory. As the correlations are, as mentioned, only moderate in comparison to the control task, we miss some deeper understanding of what more, other than these domains tested, is involved in the SPT effect. It will be important to get a better idea of the domains that can influence the performance of episodic memory for actions, possibly by enlarging the set of neuropsychological tests administered and to use more pure tests that assess a more specific kind of cognitive domains; thus allowing us to understand the more true connections between the action memory processing and the other cognitive processes known to be relatively impaired in aging (Craik & Salthouse, 2000).

The regression analysis performed separating the age groups are also not very clear in helping us to understand what could explain the SPT effect, because, when for younger adults all the tests used explain the performance in both the VT and the SPT, for older adults, no test alone, nor the set of tests taken together explained their performance in VT and SPT tasks. This can be accounted for by the fact that all the measures used to complement the Memory for Actions study were expected to show an age deficit, thus not predicting their performance in the SPT condition where they showed a better performance. However, the fact that the same happens for the VT condition may raise other questions, as their performance in this task was lower than for younger adults similar to what happens with an equivalent task (WMS Paired Associates). This may be related to the visual and sensory component present in the VT condition that is not assessed by the verbal episodic memory test WMS Paired Associates, and that could be some explanation for this finding. A limitation of this set of neuropsychological instruments used in this study is the lack of an episodic visual task, where we could more clearly compare the visual episodic system involved in the Memory for Actions test with another parallel instrument.

The results of this investigation, taken together, and although with some equivocal or weak findings, speak to some of the theoretical explanations of the SPT effect. Knowing the difficulty of associating information in a single episode inherent to the cognitive aging process (Chalfonte & Johnson, 1996; Naveh-Benjamin, 2000; Naveh-Benjamin et al., 2003), one should expect the same findings for all types of associations. However, the presence of an SPT effect for sentences that associate an object and an action, as a consequence of the implementation of that action (the enactment) unifies and strengthens the episodic action, i.e. object associations. This evidence converges with the explanation conveyed by Kormi-Nouri (1995); Kormi-Nouri & Nilsson, 1998), which refers to the potential of the SPT paradigm to increase episodic integration. The SPT condition could transform the episode into a whole unit, enhancing associative memory processing even in a population (older adults) in which this processing is generally deficient. Moreover, the comparison of this task with the WMS Paired Associates Test reinforces the evidence that binding deficits are, in fact, present in the group of older adults, and are significantly reduced in the Memory for Actions Task (SPT condition). This idea is supported by the intact source memory and the lower number of recognition errors committed under the SPT condition: older adults have declarative access to bindings made at study, and this is helped by enactment.

Other studies using the SPT paradigm, in which this pattern of results was obtained, were explained by Zimmer, Helstrup, and Engelkamp (2000) as being a reflection of the fact that the laws governing episodic memory for actions are different to the ones governing verbal episodic memory, defending the possibility of the SPT effect as being an automatic type of processing (Cohen, 1981, 1989; Cohen & Nilsson, 1988). We do not find any evidence of this here. If there is some immediate, automatic benefit, it operates also on episodic memory, in that SPT is related to tests of episodic memory, and at a later point, older adults know how they studied the information. Since we know that older people have a deficit in using strategies to encode information (e.g. Naveh-Benjamin, Brev, & Levy, 2007) it remains possible that a form of automatic and effortless encoding of information occurs at encoding, even though subsequently, standard episodic processes are those which repeat, retrieve and benefit from, this information.

Our results lend support to theories of the SPT benefit which interact with known episodic memory functions, such as the idea that there is a greater support from the semantic, sensory, and motor encoding of the object and the enacted action; the theory of multimodal coding (Bäckman & Nilsson, 1984, 1985; Engelkamp, Zimmer, & Kurbjuweit, 1995). This would be consistent with our finding that the SPT effect is related to episodic memory function, and the idea that participants can consciously report how they encoded the information. In the SPT condition, information is encoded in motor, visual and verbal codes, which appears to increase the item specificity information and also the relation to its context (Engelkamp & Seiler, 2003; Engelkamp & Zimmer, 1989), thus contributing to increased performance on this task. Consider the source task in this study: it is easier to report the source of an item if you have carried it out, since it merely gives one more piece of information about the item. In the VT condition, presumably, verbal and semantic information brings to bear on retrieval. To these cues, in the SPT condition, we can add, proprioceptive, motoric and other visuo-spatial cues not available in the VT task.

Additionally, this study suggests that the SPT effect is related to other tasks than only episodic memory, regarding the correlations between the SPT task and the executive function measures, as well as the attention and speed of processing tests, which may explain why age differences are not eliminated in this task, despite being reduced. These domains, executive function, attention and speed of processing are domains that show a decreased performance for older adults (as this study confirms), so perhaps these functions are behind the group differences on the SPT effect too. We stress, however, that the measures used here, as standardized instruments, where not pure measures of any of these domains, thus remaining the need for using more pure tasks of each cognitive domains to understand in deep their connection with the SPT effect.

In conclusion, this study finds the standard SPT effect in the context of a memory deficit in older adults. Older adults are able to report the source of items in a subsequent recognition test, showing that they retain episodic information about how they previously encountered the stimuli. Thus, we imagine that the SPT encoding, even if ‘special’ in some way, enters into the episodic system, and is later available for conscious report. This is borne out in our correlations. The general pattern is that people with high VT scores have high SPT scores, and episodic memory measures correlate with SPT scores. Thus, the SPT effect seems to act in concert with, and in line with the episodic system, and not separately from it. We continue to stress the use of this kind of rich multimodal encoding where the episodic memory is particularly impaired (Nadar & McDowd, 2008; Zimmer, 2001).
